# Growth differentiation factor 11 promotes differentiation of MSCs into endothelial‐like cells for angiogenesis

**DOI:** 10.1111/jcmm.15502

**Published:** 2020-06-25

**Authors:** Chi Zhang, Yinuo Lin, Qi Liu, Junhua He, Pingping Xiang, Dianliang Wang, Xinyang Hu, Jinghai Chen, Wei Zhu, Hong Yu

**Affiliations:** ^1^ Department of Cardiology Second Affiliated Hospital College of Medicine Zhejiang University Hangzhou China; ^2^ Cardiovascular Key Laboratory of Zhejiang Province Hangzhou China; ^3^ Department of Cardiology The First Affiliated Hospital of Wenzhou Medical University Wenzhou China; ^4^ Stem Cell and Tissue Engineering Research Laboratory PLA Rocket Force Characteristic Medical Center Beijing China

**Keywords:** angiogenesis, angiogenic therapy, differentiation, endothelial cells, GDF11, mesenchymal stem cells

## Abstract

Growth differentiation factor 11 (GDF11) is a member of the transforming growth factor‐β super family. It has multiple effects on development, physiology and diseases. However, the role of GDF11 in the development of mesenchymal stem cells (MSCs) is not clear. To explore the effects of GDF11 on the differentiation and pro‐angiogenic activities of MSCs, mouse bone marrow–derived MSCs were engineered to overexpress GDF11 (MSC^GDF11^) and their capacity for differentiation and paracrine actions were examined both in vitro and in vivo. Expression of endothelial markers CD31 and VEGFR2 at the levels of both mRNA and protein was significantly higher in MSC^GDF11^ than control MSCs (MSC^Vector^) during differentiation. More tube formation was observed in MSC^GDF11^ as compared with controls. In an in vivo angiogenesis assay with Matrigel plug, MSC^GDF11^ showed more differentiation into CD31^+^ endothelial‐like cells and better pro‐angiogenic activity as compared with MSC^Vector^. Mechanistically, the enhanced differentiation by GDF11 involved activation of extracellular‐signal‐related kinase (ERK) and eukaryotic translation initiation factor 4E (EIF4E). Inhibition of either TGF‐β receptor or ERK diminished the effect of GDF11 on MSC differentiation. In summary, our study unveils the function of GDF11 in the pro‐angiogenic activities of MSCs by enhancing endothelial differentiation via the TGFβ‐R/ERK/EIF4E pathway.

## 1 INTRODUCTION

Stem cell‐based therapy is a promising method to treat various diseases and has brought new insights into repair and regeneration of organs and tissues.^1^ Mesenchymal stem cells (MSCs) isolated from various tissues are the most widely used cells for the therapy, which are also considered as a suitable cell source for clinical treatment of cardiovascular diseases.^2^, ^3^ However, poor retention and low activity of MSCs in vivo have limited the practical use of MSC‐based therapy for ischaemic diseases. In angiogenic therapy, MSCs display multiple differentiation potentials in vitro, including the ability to differentiate into endothelial cells (ECs) and smooth muscle cells (SMCs) and can secrete various trophic factors to promote cardiovascular regeneration.^4^, ^5^

Growth differentiation factor 11 (GDF11) is a member of the transforming growth factor‐β (TGF‐β) superfamily. It is also known as bone morphogenetic protein 11 (BMP11).^6^ GDF11 is expressed in many tissues, including pancreas, intestine, kidney, skeletal muscle, heart, developing nervous system, olfactory system and retina.^7^ GDF11 plays an important role in early embryonic development and regulates the development of many organs.^8^ GDF11 signals through binding with activin type II/I receptors (ActRII/I) on cellular membrane and activates the canonical SMAD2/3 signalling pathway^9^ to realize its various biological functions.^10^, ^11^, ^12^ The activated SMAD2/3 forms complexes with universal SMAD4, then is transferred to the nucleus and regulates gene transcription. In addition to the canonical Smad signalling pathway, the TGF‐β superfamily members can also activate other non‐Smad signalling pathways.^13^, ^14^ It has been reported that GDF11 activates p38‐MAPK to regulate the size and function of the nucleolus, affects c‐Jun N‐terminal kinase (JNK) in ECs, as well as cross talking with AMPK and NF‐κB.^8^ And the extracellular regulated protein kinases (ERK) pathway was reported to be involved in the differentiation process of multi‐potent adult progenitor cells.^15^ Bone marrow‐derived MSCs have been shown to be able to promote angiogenesis by direct differentiation into ECs both in vivo and in vitro.^16^, ^17^ However, little is known about how GDF11 affects MSC differentiation and weather the effects of GDF11 on MSCs are through TGF‐β/ERK pathway.

Angiogenesis can be modulated by a number of cytokines and growth factors, among which vascular endothelial growth factor (VEGF) and TGF‐β1 play prominent roles.^18^, ^19^ VEGF and TGF‐β1 are often co‐expressed in tissues in which angiogenesis occurs, notably in a variety of tumours.^20^ TGF‐β is a multifunctional growth factor with effects on cell growth, differentiation, fibroblast activation, myofibroblast formation^21^ and ECM accumulation.^22^ Several recent studies demonstrated that TGF‐β can also induce differentiation of stem cells or progenitor cells towards smooth muscle cells or myofibroblast lineage.^23^ The plasma level of GDF11 is closely related to the formation and development of appendage skeleton^24^ and has been shown to be involved in cardiovascular disease.^10^ A recent study confirmed that higher concentration of GDF11 in the circulation was associated with a lower risk of vascular events and death, indicating that GDF11 may be a protective factor for essential in the setting of vascular events.^25^ Other studies have found that GDF11 plays an important role in angiogenesis in different organ system.^26^, ^27^, ^28^ However, there are only a few reports showing the effect of GDF11 in stem cell differentiation^29^, ^30^ and the role of GDF11 in MSCs remains to be determined.

In this study, we hypothesized that GDF11 can enhance MSC‐mediated angiogenesis by increasing the ability of MSCs to differentiate into endothelial‐like cells, as well as through its anti‐apoptosis and paracrine functions. We found that GDF11 promoted MSC differentiation into endothelial‐like cells and enhanced their pro‐angiogenic activities via activation of TGF‐β receptor and its downstream ERK/EIF4E pathway.

## 2 MATERIALS AND METHODS

### 2.1 Culture of mouse BM‐MSCs

Animal protocol was approved by Zhejiang University according to Chinese guidelines for laboratory animal care and use. Bone marrow was isolated from femurs and tibias of 8‐week‐old C57BL/6J male mice, and BM‐MSCs were obtained as described previously^31^ and cultured in DMEM medium (Hyclone, USA) supplemented with 10% foetal bovine serum (FBS) (Bioind, USA), 10 U/mL penicillin, and 10 U/mL streptomycin (Hyclone,Los Angeles, CA,USA). Cells were sub‐cultured to 80%‐90% confluence and passed after dissociation with 0.25% Trypsin & 0.02% EDTA (Genom, China) (Figure S1A). For normal oxygen conditions (21% O2, 5% CO2, 37℃), cells were incubated in a standard humidified CO_2_ incubator. All experiments were performed using cells at passage between 3 and 5.

### 2.2 Characterization of MSCs

MSCs were characterized by flow cytometric analysis of surface markers and were positive for: CD44, CD105, CD90; negative for: CD31, CD45(Figure S1B). At room temperature, cells were dissociated, re‐suspended in phosphate‐buffered saline (PBS) and incubated with antibodies against following markers in dark for 30 mins: CD44 (Ebioscience, Santiago, USA, Cat #12044181), CD105 (Ebioscience,USA, 550546), CD90 (Ebioscience,553004), CD31(Ebioscience, 561073) and CD45 (Ebioscience, USA, 553098). Non‐specific mouse IgG‐APC and IgG‐APC (Ebioscience,USA) were used as controls. After incubation, the cells were washed twice with PBS and analysed by flow cytometry (BD Biosciences,New Jersey, USA). The data were analysed by Flowjo software.

The ability of MSCs to differentiate into osteocytes and adipocytes was examined by culturing MSCs at passage 3 with osteogenic medium: DMEM supplemented with 10% FBS, 10 mmol/L β‐glycerophosphate, 50 μmol/L ascorbate‐2‐phosphate and 0.1 μmol/L dexamethasone (all from Sigma‐Aldrich), or adipogenic medium: DMEM supplemented with 10%FBS, 5 μg/mL insulin, 5 mmol/L isobutyl methylxanthine, 60 μmol/L indomethacin, and 1 μmol/L dexamethasone (all from Sigma‐Aldrich) for 2 weeks. Medium was changed every 3 days. After 2 weeks, cells were stained with Alizarin Red S (Solarbio, Life science) or Oil red O (Solarbio, Life science) to detect osteocytes and adipocytes, respectively (Figure S1C).

### 2.3 Lentiviral vector transduction

Lentiviral vectors carrying genes for GDF11 and GFP (LV‐GDF11‐GFP) or control vectors (LV‐GFP) and Luciferase (LV‐ Luc) were prepared by Genechem (Shanghai, China). MSCs were seeded at 1x10^5^ cell per well onto 12‐well plates one day before transduction. Medium was changed with fresh serum‐free DMEM medium (500 μL/well), and viral vectors (~2.5 μL) premixed with 20 μL HiTransG P transfection reagent (Genechem) were added to each well to reach a multiplicity of infection (MOI) at 50 for all transduction. Culture medium was changed 12 hours after transfection with DMEM containing 10% FBS. After 48 hours, cells were observed under fluorescent microscope for GFP^+^ cells (Figure S2A). Then, the successfully transduced cells were selected by culturing the cells in the presence of purinomycin. Expression of GDF11 at levels of mRNA and protein was detected by RT‐PCR (Figure S2B) and Western blot (Figure S2C), respectively. The cells transducted LV‐GDF11‐GFP/ Luc were abbreviated as MSC^GDF11^, and control vectors (LV‐GFP/ Luc) were abbreviated as MSC^vector^. This abbreviation is used the following text.

### 2.4 Endothelial cell differentiation

MSCs were cultured in 12‐well plates (1 × 10^5^ cells/well) with DMEM medium for 24 hours at 37°C and then cultured in M199 medium (Corning, USA) supplemented with 2% FBS, 50 ng/mL VEGF165 (PEPROTECH, USA, #450‐32‐10UG) for 14 days. Medium was changed every 2 days. Cells were detached for analysis of surface markers CD31 (Ebioscience, 561073), VEGFR2, (Ebioscience, 561993) by flow cytometry as described above, or cellular protein was extracted for Western blot analysis.

For mechanism study, inhibitor of TGF‐β receptor (LY2109761) (SELLECK, USA) or of ERK1/2 (SD5978) (Huaan, China), both in a final concentration of 10 µmol/L, or DMSO(1 μL/ml) was added into the fresh medium for 45 minutes prior to the addition of VEGF165 for differentiation culture for 14 days.

### 2.5 Western blot assay

Cell lysates were prepared using radioimmunoprecipitation assay (RIPA) buffer (Beyotime, China, P0013B). Total protein was quantified by BCA protein assay (Bio‐Rad, Berkery). Each sample was adjusted to equal amount of protein using 5X loading buffer for loading. The samples were separated by SDS‐PAGE, transferred to a polyvinylidene fluoride membrane, and immunoblotted with the following antibodies: GDF11 (DGDF80, R&D Systems,Emeryville, CA,USA), HGF (ab83760, Abcam, USA), VEGFR1 (ET1605‐11,Huabio，China), CD31 (#77699, Cell Signalling TechnologyBoston,MA, USA), VEGFR2 (#9698, following Abs are all from Cell Signalling Technology,USA), phospho‐p44/42 (Thr202/Try204 phospho‐ERK1/2,#4370), p44/42 MAPK (Erk1/2,#4696), BCL2 (#2827), BAX (#14796), Cleaved Caspase3 (#9661), phospho‐Smad2 (Ser465/Ser467,#18338), phospho‐Smad3 (Ser423/425, # 9520), Smad2(#5339), Smad3(#9523), anti‐β‐actin (#3700), EIF4E (R1512‐8,Huabio, China), Phospho‐eIF4E (S209) (ET1608‐66,Huabio, China) and VEGF ( ER30607,Huabio, China),at 4°C overnight. After incubation of the membranes with peroxidase‐conjugated secondary antibodies (Cell Signalling Technology,USA), bands were visualized using enhanced chemiluminescence reagents (Bio‐Rad,Los Angeles, CA,USA).

### 2.6 Real‐time RT‐PCR

Total RNA was extracted using Trizol reagent (Invitrogen, USA) according to the manufacturer's protocol. Total RNA (1 μg) was used for reverse transcription to synthesize cDNA using PrimeScript™ 1st Strand cDNA Synthesis Kit (TaKaRa, Dalian, China), and SYBR^®^ Premix Ex TaqTM II (Tli RNaseH Plus) (TaKaRa) was applied for real‐time RT‐PCR process on an ABI PRISM 7500 Fast Detection System (Applied Biosystems, Carlsbad, CA, USA) according to the standard method. Each sample was performed in triplicated and all results were normalized to the expression of normalized to β‐actin (ACTIN). Fold expression relative to the reference ACTIN gene was calculated using the comparative method 2^−ΔCt^. The sequences of PCR primers were listed in Table S1.

### 2.7 Immunohistochemistry staining

Cells were washed with PBS containing 3% bovine serum albumin (BSA) and fixed with 4% paraformaldehyde for 15 minutes. The cells were then permeabilized with 0.5% Triton X‐100 for 10 minutes, blocked with 3% BSA in PBS for 30 minutes at room temperature and then incubated with primary antibody CD31 (#77699, Cell Signalling Technology) and VEGFR2 (#9698, Cell Signalling Technology), overnight at 4°C, followed by incubation with secondary antibodies for 1 hour at 37°C. Nuclei were stained with Hoechst (Thermo Fisher, 33342) for 5 minutes. The cells were then washed three times and viewed using a fluorescence microscope (Leica, Germany).

### 2.8 Tube formation assay

Tube formation assay was performed according to the manufacturer's protocol. Matrigel (50 µL) (Corning, New York, USA, #356231#) was added to each well of a 96‐well plate and allowed to polymerize. GFP‐transduced MSCs (1 × 10^4^ cells) were suspended in culture medium as mentioned above containing 2% FBS and plated on Matrigel. After culture for 2‐12 hours, images were taken using a fluorescence microscope (Leica,Germany). The tube formation was quantified by analysing the total tube length in each well with Image‐Pro Plus (MediaCybernetics, USA).

### 2.9 Cell viability assay

For in vitro cell viability assay, GDF11‐overexpressed and negative control—MSCs were plated on collagen‐coated 96‐well plates (2 × 10^3^ cells/well) and cultured in serum‐free medium under hypoxia (0.1% O_2_, 5% CO_2_) at 37°C for 48 hours. Then, 10 μL of Cell Counting Kit‐8 (CCK‐8, Dojindo, Japan) was added and incubated for 2 hours at 37°C, and the absorbance was determined at a wavelength of 450 nm. MSC viability was evaluated using OD value as described above.

### 2.10 Matrigel Plug Assay in vivo

Male C57BL/6 mice (8‐week‐old, weighting 22‐25 g) were used. MSCs transduced with GDF11 vector (MSCs^GDF11^) or with control vector (MSCs^Vector^) (each 5x10^6^ cells) were suspended in 200 µL PBS, mixed with 200 µL Matrigel (Corning, #354262#) in a 1 mL syringe and subcutaneously injected into inguinal region of mice using 26 gauge needle. After 10 days, Matrigel tissues were isolated and half were dissolved with Cell Recovery Solution (Corning, #354253#) and then analysed by Flow Cytometry, and the rest of plug was fixed with 10% formalin overnight and embedded in paraffin for histological analysis.

### 2.11 Histological analyses

To examine the capillary and arteriole densities, paraffin sections of Matrigel were stained with following antibodies: rat anti‐mouse CD31 (562939, BD Bioscience), rat anti‐rabbit luciferase (ab185924, Abcam) and rat anti‐rabbit GFP (ab290, Abcam). Alexa Fluor 488 or 550 conjugated antibody (Invitrogen) were used for secondary staining. After being mounted with Hochest mounting medium, the samples were analysed using a fluorescence confocal microscope (Leica). For morphometric analysis, sections were stained with haematoxylin and eosin (H & E). Images were taken under 200/400× magnification.

### 2.12 Transfection of siRNA

Small interfering RNA (siRNA) specific for GDF11 gene expression (siRNA^GDF11^) and control scrambled siRNA were synthesized by GenePharma Co., Ltd (Shanghai, China). Cells were seeded at 1x10^5^ per cell into 12‐well plates one day before transfection. Lipofectamine 2000 (Invitrogen, Carlsbad, CA, USA) (2 μL/mL) was mixed with siRNAs (final 50 nmol/L) in reduced serum medium OptiMEM (Invitrogen) and then added into each well (1ml per well). Medium was changed to DMEM containing 10% FBS 8 hours after transfection and cultured for further analysis. Expression of GDF11 at levels of mRNA and protein was detected by RT‐PCR and Western blot, respectively. For the experiment of differentiation that lasted 14 days, the cells were transfected with siRNA again 7 days after the first transfection to maintain GDF11 being knocked down. VEGF165 was added to the culture 24 hours after either transfection to induce differentiation of MSCs to ECs.

### 2.13 Cell apoptosis analysis

For in vitro cell apoptosis assay, MSCs^GDF11^ and MSCs^Vector^ were plated on 24‐well plates (2 × 10^4^ cells/well) and cultured in serum‐free medium under hypoxia (0.1% O_2_, 5% CO_2_) at 37°C for 48 hours. After 48 hours, cell apoptosis was analysed using a TUNEL Cell Apoptosis Assay Kit (Beyotime,Shanghai, China) according to the manufacturer's instructions. Briefly, cells on plates were washed twice with PBS and fixed with 4% paraformaldehyde for 15 minutes, followed with PBS wash twice. Then, cells were incubated with TUNEL reagent for 1 hour. Nuclei were stained with Hoechst for 5 mins. The cells were then washed three times and viewed using a fluorescence microscope.

### 2.14 Statistics analysis

Results were expressed as means ± SD (standard deviation). Continuous variables were compared by Student's t test, and multiple comparisons were performed by one‐way ANOVA with a Bonferroni correction. Statistical analyses were performed using Prism 6 (GraphPad Software Inc,San Diego, CA, USA). A value of *P* < 0.05 was accepted as statistically significant.

## 3 RESULTS

### 3.1 Mutual effect of GDF11 and VEGF during MSC differentiation

**FIGURE 1:**
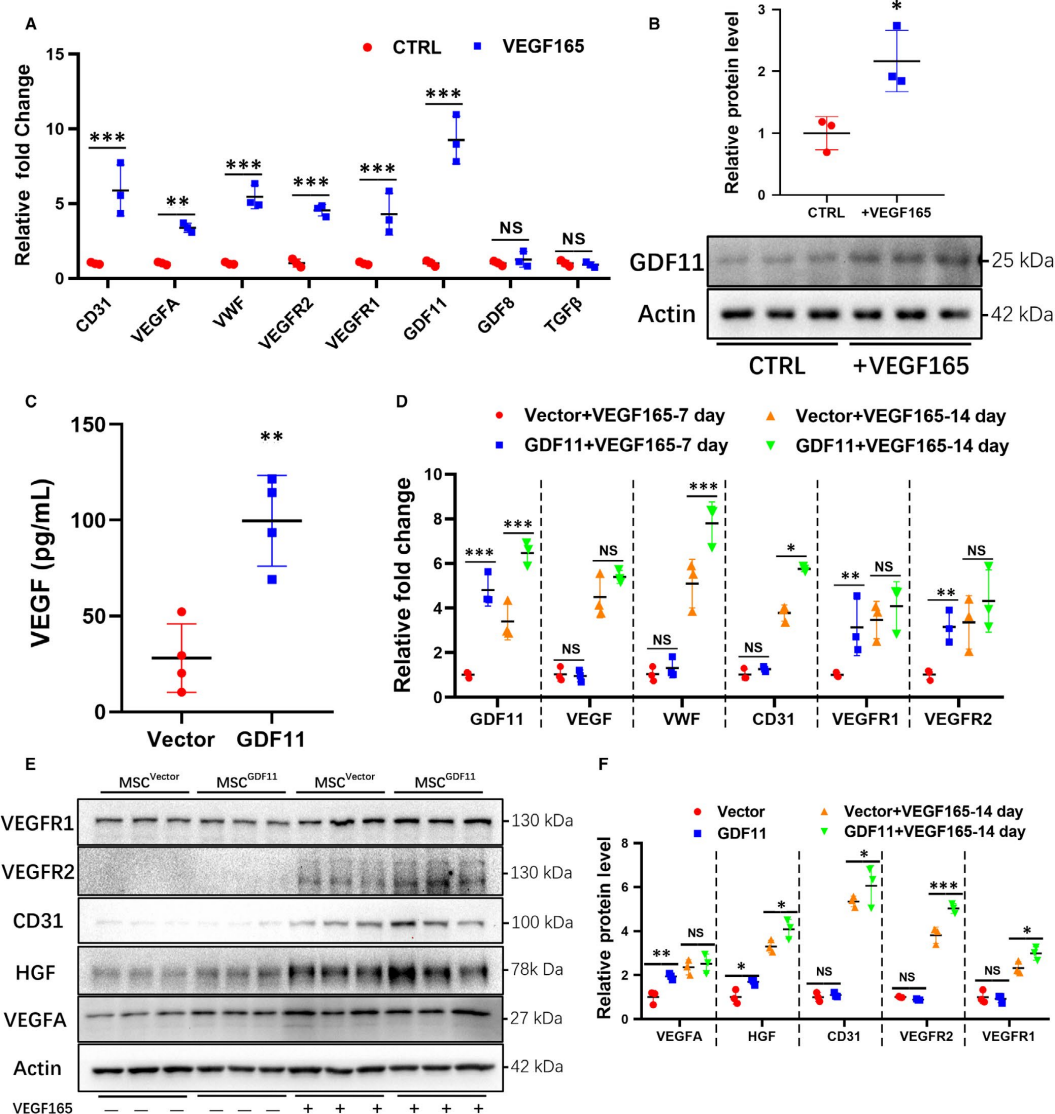
Role of GDF11 in the differentiation of MSCs into endothelial‐like cells. MSCs were cultured in the absence (Control) or presence of VEGF165 (50 ng/mL) for 14 d to induce endothelial differentiation. A, mRNA levels of various genes in MSCs were analysed by RT‐PCR (n = 3). Data are expressed as fold change relative to control. B, GDF11 protein was analysed by Western blot (n = 3). C, Detection of VEGF in the supernatants of MSC^Vector^ (Vector)or MSC^GDF11^(GDF11) after 1‐day culture (n = 4). D, mRNA levels in MSC^Vector^ or MSC^GDF11^ after the cells were cultured for 7‐ or 14 d with or without VEGF165 (n = 3). E, Proteins in MSC^Vector^(Vector) or MSC^GDF11^(GDF11) were analysed by Western blot before and after culture with VEGF165 for 14 d (n = 3). Molecular weights are indicated next to blots. F, Quantification of proteins in E. Data are presented as the mean ± SD for at least 3 independent experiments and were analysed. *P < 0.05; **P < 0.01 and ***P < 0.001. HGF, hepatocyte growth factor; VEGF, vascular endothelial growth factor; PECAM‐1/CD31, Platelet endothelial cell adhesion molecule‐1; VEGFR1 and VEGFR2, VEGF receptor 1 or 2, respectively

**FIGURE 2:**
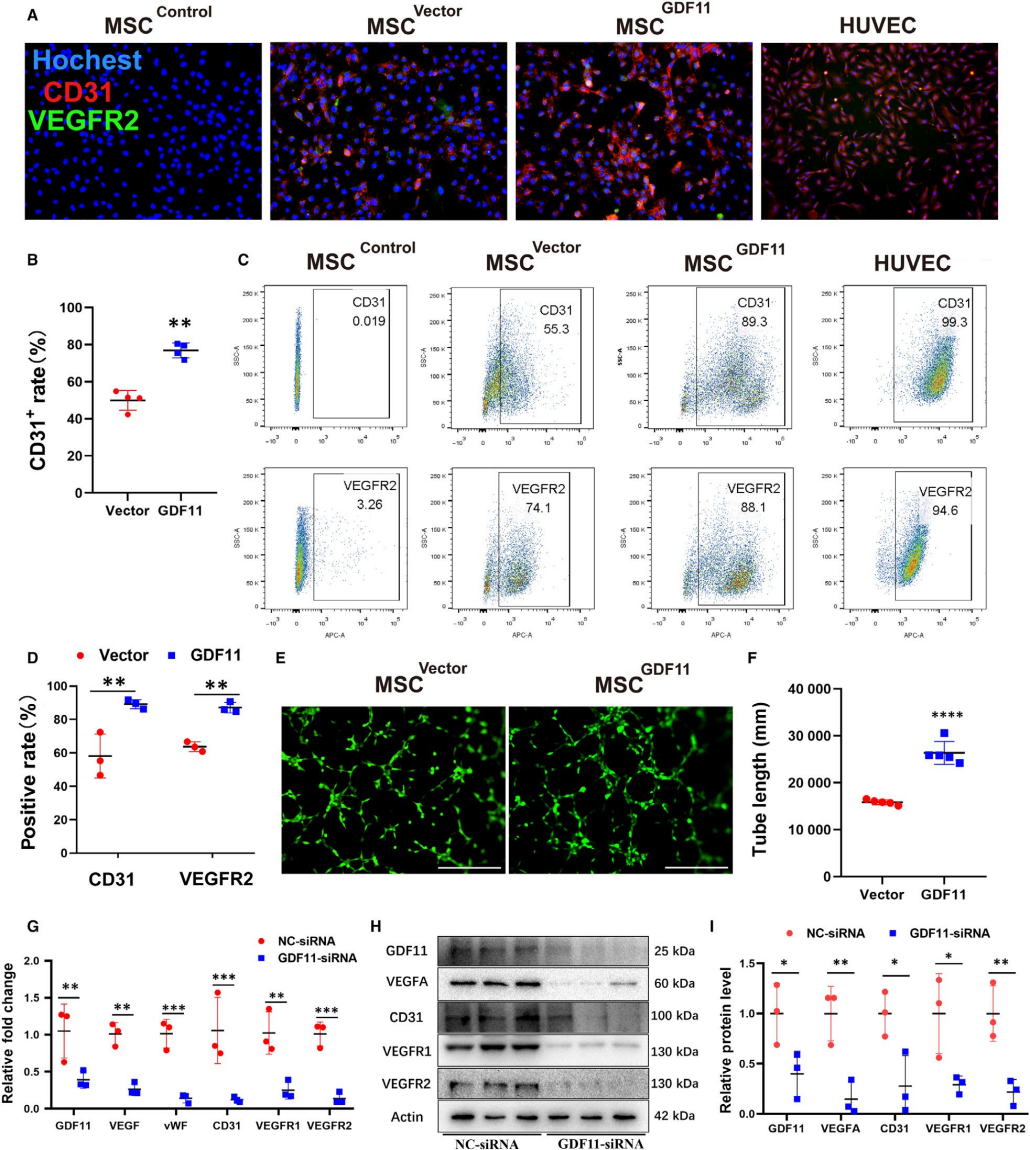
The effect of GDF11 in differentiation of MSCs induced with VEGF165. A, Immunofluorescent staining of MSC^Vector^ (2nd raw), MSC^GDF11^ (3rd raw) with Abs against EC marker CD31 (red) and VEGFR2 (green). Nucleus was stained with DAPI (blue). MSCs were cultured at the presence of VEGF165 for 14 d to induce differentiation. Scale bars = 100 μm. HUVEC was used as positive control (3rd raw), MSC^control^ (1st raw) were cultured without VEGF165, and used as a negative control. B, Quantification of CD31+ cells in MSC^Vector^ and MSC^GDF11^ shown in A. C, Flow cytometry analysis of EC markers (CD31 in upper panel, VEGFR2 in lower panel) on MSC^GDF11^ and MSC^Vector^. And HUVEC was used as positive control, MSC^control^ without VEGF induction were used as negative control. D, Quantification of positive rates in C (n = 3). E, Representative images showing tube formation of MSCs after differentiation for 14 d on Matrigel for 2 or 8 h. Scale bars: 200 μm. F, Quantification of tube formation in E by measuring branch lengths of formed tube for 8 h. Only length > 200 μm was counted (n = 5 in each group). Data are presented as the mean ± SD for at least 3 independent experiments. G, mRNA levels were examined by RT‐PCR after cultured at the presence of VEGF165 for 14 days to induce differentiation for MSCs either transfected with NC‐siRNA (control) or GDF11‐siRNA (n = 3). H) Western blot assay for detection of proteins: GDF11, VEGFA, CD31, VEGFR1 and VEGFR2 after siRNA transfection and cultured with VEGF165 for 14 days. I, Quantitative analysis of H. *P < 0.05; **P < 0.01 and ***P < 0.001

First, we examined gene expression in MSCs under conditions promoting endothelial differentiation. Expression of GDF11 along with other EC markers CD31, VEGFA, VWF, VEGFR2 and PDGFR was significantly increased after MSCs were cultured with VEGF165 for 14 days, while GDF8 and TGF‐β were not changed (Figure 1A,B). On the other hand, when GDF11 was overexpressed in MSCs by transduction with lentiviral vector (MSC^GDF11^), significantly more VEGF was secreted into supernatant as compared with control MSC^Vector^ (Figure 1C), while no significant change in mRNA level was observed (Figure S3A‐B). After MSC^GDF11^ were induced for endothelial differentiation with VEGF165 for either 7‐ or 14‐days (Figure S4A), higher expression of EC markers was detected as compared with control MSC^Vector^ (Figure 1D‐F). These phenomena were further confirmed by immunofluorescent staining (Figure 2A,B and Figure S4B,C) and flow cytometry (Figure 2C,D). More CD31^+^ and VEGFR2^+^ cells were observed in MSC^GDF11^ than MSC^Vector^. Furthermore, such differentiated MSC^GDF11^ demonstrated higher capability to form tube‐like structures in Matrigel assay than MSC^Vector^ (Figure 2E,F). These data indicated that GDF11 was up‐regulated during endothelial differentiation of MSCs and, in turn, that GDF11 enhanced MSC differentiation into endothelial‐like cells.

### 3.2 Lower expression of GDF11 in MSCs reduces their differentiation into endothelial‐like cells

To confirm the effect of GDF11 on MSCs, siRNA specific for GDF11 gene was transfected into MSCs to knock down GDF11 expression. GDF11 at both mRNA (Figure 2G) and protein levels (Figure 2H,I) in the GDF11‐siRNA‐treated MSCs was lower than that in the NC‐siRNA‐treated control. After MSCs were induced with VEGF165 for endothelial differentiation for 14‐days, lower expression of EC markers was detected in the GDF11‐siRNA‐transfected MSCs as compared with the NC‐siRNA control (Figure 2G‐I; and Figure S5). Along with the down‐regulation of GDF11, the mRNA expressions of genes for VEGFA, vWF, CD31, VEGFR1 and VEGFR2 appeared to be reduced (Figure 2G). The respective proteins in GDF11‐siRNA group were also significantly decreased (Figure 2H,I). These results were further confirmed by flow cytometry analysis (Figure S5A,C) and immunofluorescent staining (Figure S5B,D).

### 3.3 GDF11 enhances survival rate of MSCs and protects MSCs from hypoxia‐induced apoptosis in vitro

**FIGURE 3:**
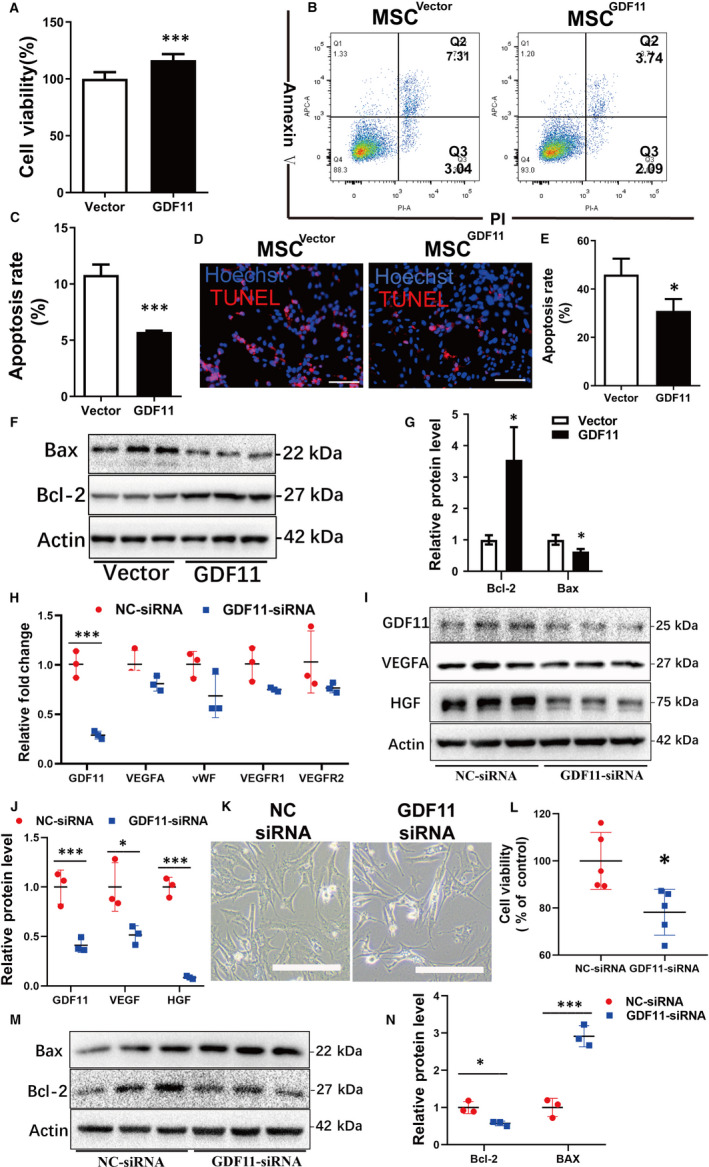
Effects of GDF11 in viability of MSCs in vitro. MSCs were cultured under serum deprivation and hypoxic conditions (0.1**%** O_2_) for 48 h to induce apoptosis in vitro. A, Viability of MSCs was examined by cell counting kit‐8 (CCK‐8) assay (n = 5). B, Apoptotic cells were detected with flow cytometry assay after staining with Annexin V & PI. C, Quantifications of apoptotic cells by adding Q2 and Q3 in B. D, TUNEL assay of apoptotic MSCs (pink). Nuclear were stained with Hoechst (blue). E, Quantifications of apoptotic MSCs in D (n = 3 in each group). F, Apoptosis‐related proteins B‐cell lymphoma‐2 protein (BCL‐2) and Bcl2 Associated X Protein (BAX) were detected by Western blot. G, Quantifications of BCL‐2 and BAX related to β‐Actin (n = 3). H, mRNA levels were examined by RT‐PCR for MSCs either transfected with NC‐siRNA (control) or GDF11‐siRNA (n = 3). I, Western blot assay for detection of proteins: GDF11, VEGFA and HGF after siRNA transfection. J, Quantitative analysis of I. K, Representative images of the cell morphology after transfected with specified siRNA for 24 h. Scale bars: 200 μm. L, Viability of MSCs was examined by CCK‐8 assay after knock‐down of GDF11 by siRNA (n = 5). M, Western blot assay for detection of apoptosis‐related proteins BCL‐2 and BAX after siRNA transfection. N, Quantitative analysis of M. Data are presented as mean ± SD for at least 3 independent experiments. *P < 0.05; **P < 0.01 and ***P < 0.001

To mimic the ischaemic environment in vivo, MSCs were exposed in hypoxic environment for 48 hours to induce apoptosis in vitro. Higher viability of MSC^GDF11^ was observed as compared with control MSC^Vector^ by using CCK‐8 assay (Figure 3A). Less apoptotic cells in MSC^GDF11^ than in control MSC^Vector^ were detected by flow cytometry (Figure 3B,C) and TUNEL assay (Figure 3D,E) after treatment with hypoxia, which was confirmed with Western Blot analysis of apoptosis‐related proteins (Figure 3F,G).

Likewise, knock‐down of GDF11 expression in MSCs with siRNA reversed the effects of GDF. The mRNA levels of VEGFA, vWF, VEGFR1 genes appeared to be lower in trend in the GDF11‐siRNA‐treated MSCs in comparison with the NC‐siRNA transfected control (Figure 3H). VEGFA and HGF proteins were also significantly decreased in the GDF11‐siRNA‐treated MSCs (Figure 3I,J). The cell morphology became unhealthier as compared with NC‐siRNA transfected cells: more bright detaching cells were observed after the down‐regulation of GDF11 and exposed to hypoxic condition (Figure 3K). Cell viability was also lowered in GDF11‐siRNA transfected MSCs than the control (Figure 3L) The effect of GDF11 on apoptosis‐related proteins Bax and Bcl‐2 was also reversed by the siRNA (Figure 3M,N).

### 3.4 GDF11 facilitated MSC‐mediate angiogenesis in vivo

**FIGURE 4:**
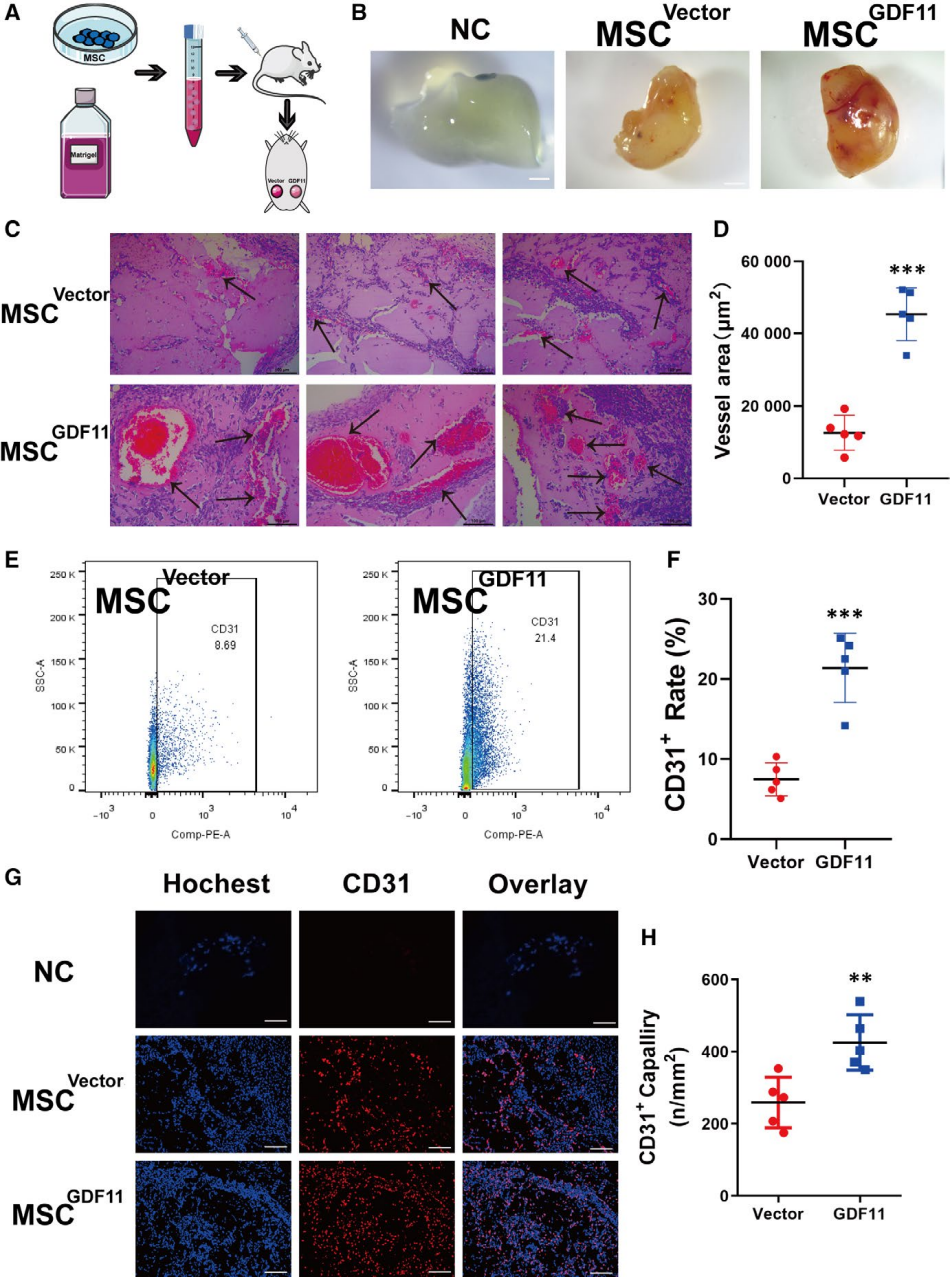
MSC^GDF11^ promoted angiogenesis in Matrigel plugs in vivo. A, Scheme of angiogenesis assay in vivo using Matrigel plugs. The Matrigel plugs mixed with MSCs^Vector^ or MSCs^GDF11^ were implanted subcutaneously into the left or right side, respectively, of inguinal region of mice for 10 d. B, Photographs of the recovered Matrigel plugs showing gross appearance of angiogenesis (n = 5). NC: PBS with no cells as Negative control, Vector: MSCs^Vector^, GDF11: MSCs^GDF11^, at 10x magnification. Scale bars: 2 mm. C, Representative images of H & E staining of plug sections. Arrows indicate vessels. Scale bars: 100 μm. D, Quantification of vessel density by measuring the areas. E, Representative pictures of flow cytometry analysis of CD31^+^ endothelial‐like cells in the recovered Matrigel plugs. F, Quantification of CD31^+^ endothelial‐like cells in E. G, Representative images of sections of recovered Matrigel plugs immune stained with antibodies against CD31 (red). Nuclei were stained with DAPI (blue). Scale bars: 100μm. H, Quantification of CD31^+^ EC density in e. Data are presented as the mean ± SD for at 2 independent experiments and were analysed. *P < 0.05, **P < 0.01, ***P < 0.001

In order to verify the pro‐angiogenic activity of GDF11 in vivo, Matrigel plugs containing MSC^Vector^ or MSC^GDF11^ were implanted into mice (Figure 4A). The recovered Matrigel plugs containing MSC^GDF11^ appeared more reddish indicating more vessel formation in the plug allowing more red blood cells come in as compared with those treated with MSC^Vector^ (Figure 4B and Figure S6A). Indeed, more blood vessels were observed in sections of MSCs^GDF11^ plugs as compared to those from MSC^Vector^ controls (Figure 4C,D). In addition, more CD31^+^ endothelial‐like cells were detected by flow cytometry from MSC^GDF11^‐plugs (21.40 ± 2.059%) as compared to than that of MSC^Vector^ controls (7.478 ± 4.323%, n = 5) (Figure 4E,F). Capillary density as detected by immunostaining for CD31 was also significantly higher in plugs with MSCs^GDF11^ as compared with MSC^Vector^ controls (Figure 4G,H).

### 3.5 GDF11 promoted MSC differentiation into endothelial‐like cells in vivo

**FIGURE 5:**
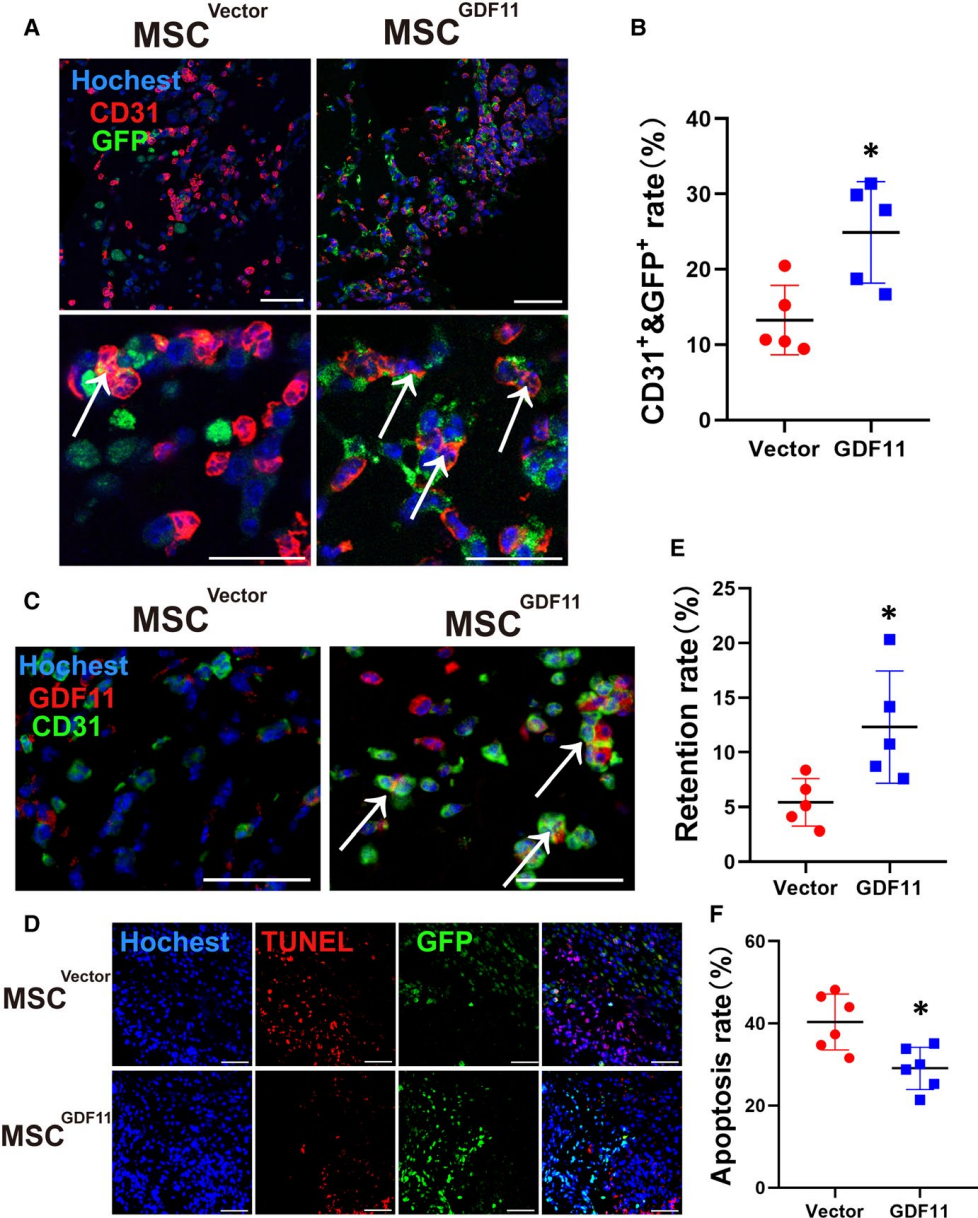
Effect of GDF11 on MSC differentiation, retention and apoptosis in vivo. MSC^GDF11^ or MSCs^Vector^ expressing GFP or luciferase were mixed with Matrigel and implanted into mice. Paraffin sections of the recovered plugs 10 d after implantation were stained with DAPI for nuclei (blue) and specified antibodies (n = 5). A, Antibodies against GFP (green) and CD31 (red) were used for detection of endothelial‐like cells. White arrows point at the MSCs (orange) whose GFP was colocalized with CD31. The pictures in upper panel were taken at 400× magnification and the lower panel are magnified 2×. Scale bars: 50 μm. B, Rate of MSC differentiation into endothelial‐like cells was quantified by dividing number of double positive cells by number of total retained MSCs in an image. C, Antibodies against GDF11 (red) and CD31 (green) for endothelial‐like cells were used. White arrows point at the double positive cells (orange). The pictures were taken at 600× magnification. Scale bars: 50 μm. D, Sections were stained for TUNEL to identify apoptotic cells (red) and retained MSCs (GFP). Scale bars: 100 μm. E, Retention rates were quantified as the number of GFP+ cells out of the total number of cells. F, Apoptosis rate was quantified by the percentage of cells positive for TUNEL staining. Data are presented as the mean ± SD for at 2 independent experiments and were analysed. *P < 0.05; **P < 0.01 and ***P < 0.001

To track the fate of implanted MSCs in the Matrigel plug, both MSCs^GDF11^ and MSC^Vector^ were transduced with genes for GFP or luciferase before implantation. Immunofluorescence co‐localization analysis (Figure 5A) was performed to examine the implanted MSCs in the plugs recovered 10 days after implantation. There were more cells that were positive for both GFP and CD31 in plugs containing MSCs^GDF11^ as compared to those with MSC^Vector^ (Figure 5B). This was further confirmed by co‐localization of luciferase^+^ with CD31^+^ cells (Figure S6B,C). And we did the double‐staining with antibodies against GDF11 and CD31. More CD31^+^ cells in the MSC^GDF11^ group were observed (Figure 5C). These results indicate that the presence of GDF11 in MSCs^GDF11^ augments their ability to differentiate into CD31^+^ endothelial‐like cells.

Furthermore, there were more MSCs^GDF11^ than control MSCs^Vector^ in the plugs recovered 10 days after implantation (Figure 5D). The retention rate was higher for MSCs^GDF11^ than MSCs^Vector^ (Figure 5E) concomitant with less apoptosis (Figure 5F). The enhanced survival rate of MSCs^GDF11^ versus MSCs^Vector^ was further confirmed in vitro when MSCs were placed in a hypoxic environment for 48 hours to induce apoptosis (Figure S7A); in addition, fewer TUNEL^+^ cells were observed in the MSCs^GDF11^ as compared to MSCs^Vector^ (Figure S7B).

### 3.6 Effects of GDF11 involve TGF‐β receptor‐mediated ERK/EIF4E signalling pathway

**FIGURE 6:**
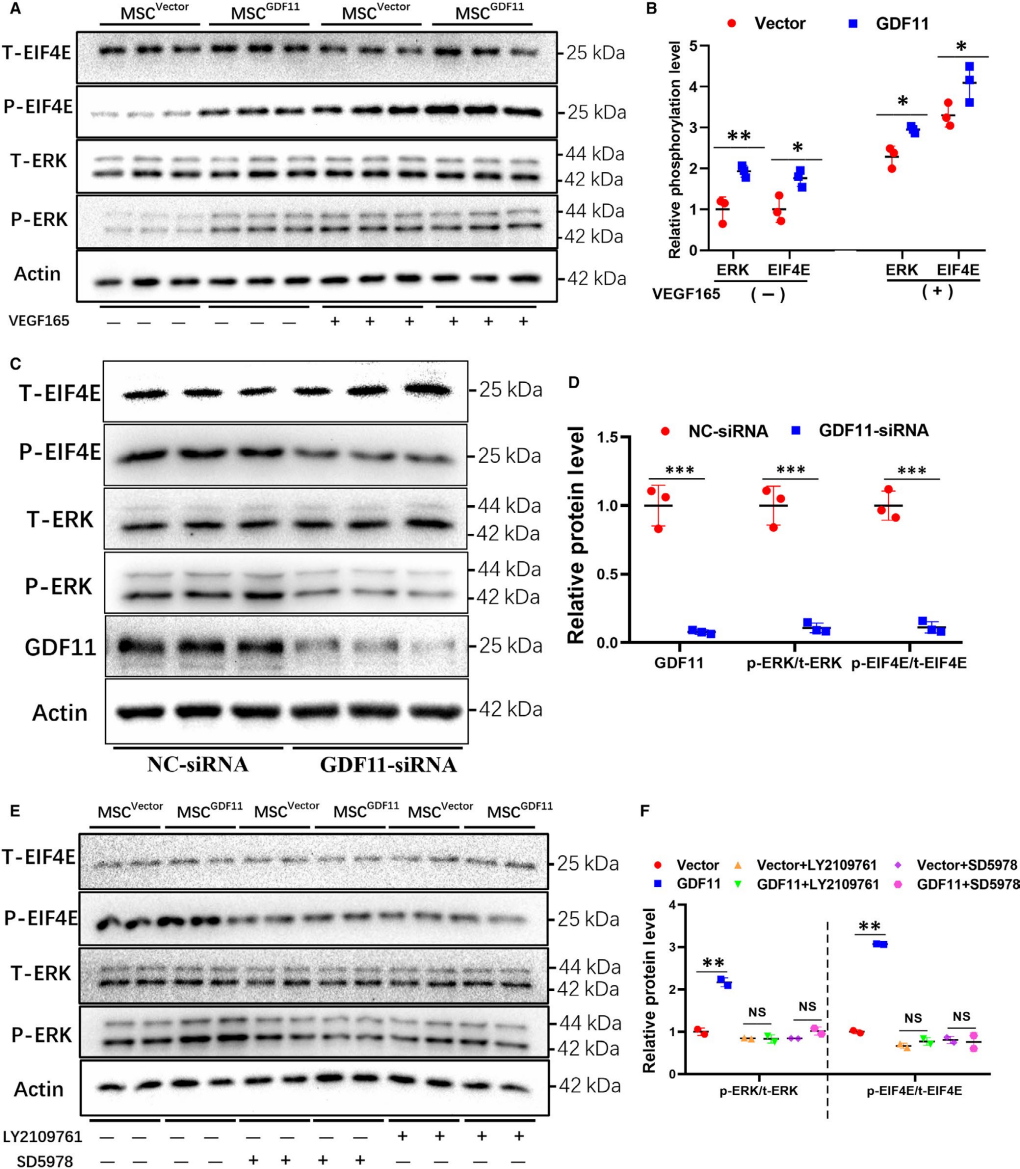
Analysis of the molecular pathways underlying GDF11‐induced MSC differentiation. A, MSCs^Vector^ and MSC^GDF11^ were cultured in the presence or absence of VEGF165 for 14days. Total (T) and phosphorylated (P) ERK and EIF4E were examined by Western blot (n = 3). B, Quantitative analysis of A. The density of each band was calibrated with its corresponding Actin band. Relative phosphorylation levels were quantified by dividing P over T, which was then compared with the level in control MSCs^Vector^ to set as 1. C, MSCs were transfected with control (NC)‐ or GDF11‐specific siRNA. Total (T) and phosphorylated (P) ERK and EIF4E were examined by Western blot (n = 3). D, Quantitative analysis of C in the same way as B. E, MSCs^Vector^ and MSC^GDF11^ were cultured with VEGF165 for EC differentiation in the presence or absence of inhibitors for TGF‐β receptor (LY2109761) or for ERK (SD5978) for 14days. T‐ and P‐ ERK and EIF4E were examined by Western blot. F, Quantitative analysis of E. Data are presented as the mean ± SD. *P < 0.05; **P < 0.01 and ***P < 0.001. Each WB was repeated for at least 2 times

**FIGURE 7:**
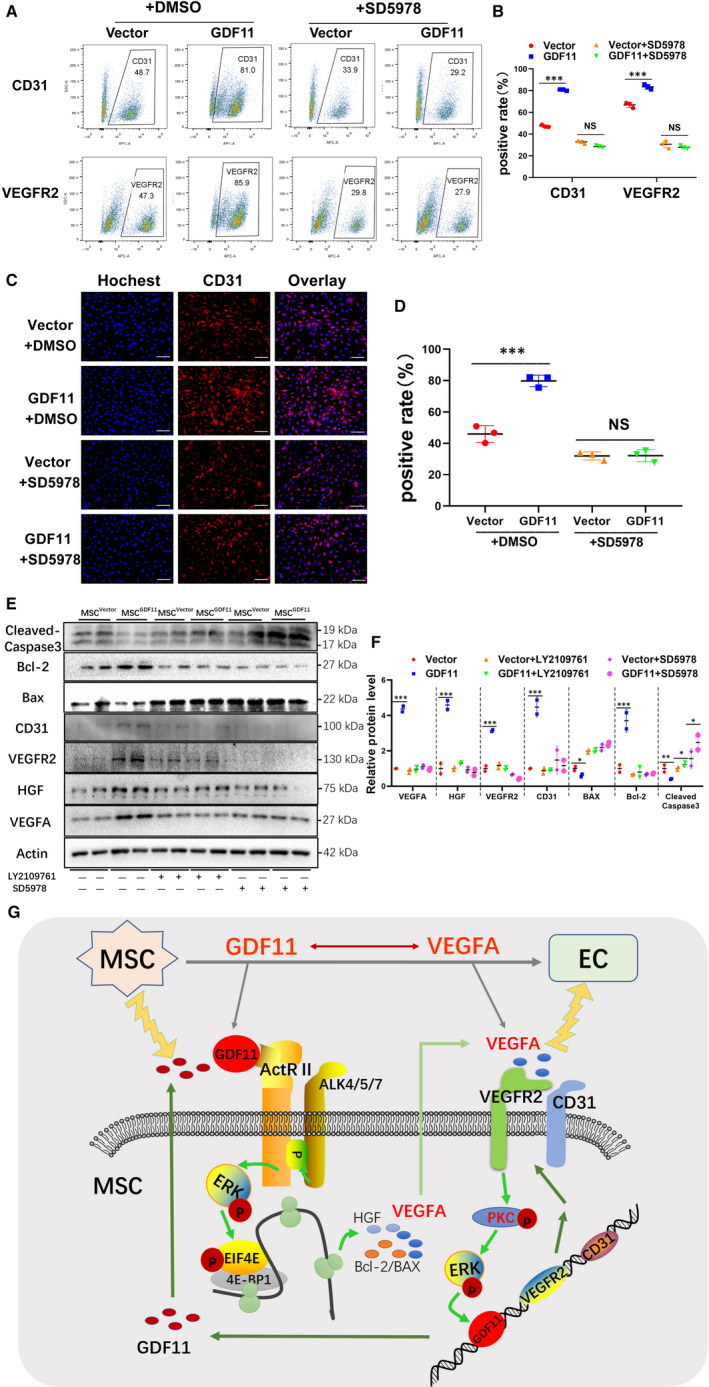
Effect of GDF11 on the differentiation of MSCs into endothelial‐like cells was blocked by ERK inhibitor. MSC^Vector^ and MSC^GDF11^ were treated with inhibitors for ERK (SD5978) or for TGF‐β receptor (LY2109761) or with DMSO as a control during the differentiation of MSCs into endothelial‐like cells for 14 d. A, Representative results of flow cytometry analysis of MSC^Vector^ (Vector) and MSC^GDF11^ (GDF11) for their expression of CD31 and VEGFR2. B, Quantitative analysis of A (n = 3). C, Immunofluorescent staining of MSC^Vector^ and MSC^GDF11^ with antibody against CD31 (red) after culture with or without ERK inhibitor. Nucleus was stained with Hochest. Scale bars = 100 μm. D, Quantitative analysis of C (n = 3). CD31^+^ rate was obtained by dividing CD31^+^ cells (red) by total number of cells (blue) in a picture. E, Western blot analysis of proteins in MSC^Vector^ and MSC^GDF11^ in the presence of absence of inhibitors LY2109761 or SD5978during the differentiation of MSCs into endothelial‐like cells. F, Quantitative analysis of E (n = 2). Each WB was repeated for at least 2 times. G, Schematic diagram of the proposed mechanisms by which GDF11 promotes MSC differentiation into endothelial‐like cells. GDF11 interacts with TGF‐β receptor, resulting in activation of the ERK/EIF4E pathway, which enhances the expression of proteins for angiogenesis and anti‐apoptosis, and augment of EC markers during cultured with VEGFA. Data are presented as the mean ± SD. *P < 0.05; **P < 0.01 and ***P < 0.001. BCL‐2 for B‐cell lymphoma‐2 protein; BAX for Bcl2 Associated X Protein

To determine the molecular mechanism underlying in the effects of GDF11 on MSCs, we examined ERK1/2 signalling pathway, which was reported to be involved in the differentiation process of progenitor cells.^15^ When GDF11 was overexpressed in MSCs, phosphorylation of both ERK and EIF4E was significantly increased (Figure 6A,B). Similarly, when MSCs were cultured under differentiation conditions with VEGF165, both ERK and EIF4E were activated; overexpression of GDF11 further enhanced their phosphorylation. In the meantime, siRNA‐mediated knock‐down of GDF11 in MSCs led to a substantial decrease in phosphorylation of both ERK and EIF4E (Figure 6C,D). To explore whether ERK and EIF4E were essential for GDF11‐induced endothelial‐like cells differentiation, LY2109761, an inhibitor of TGF‐β receptor or SD5978, an inhibitor of ERK1/2 was added to the culture in course of differentiation. Indeed, phosphorylation of both ERK and EIF4E was blocked by either inhibitor (Figure 6E,F). Furthermore, treatment with the inhibitors also blocked the differentiation of MSCs into endothelial‐like cells. GDF11‐induced expressions of EC markers CD31 and VEGFR2 were decreased to the similar level as the control when ERK inhibitor was added into the differentiation medium detected by flow cytometry (Figure 7A,B) and immunofluorescent staining (Figure 7C,D). These were confirmed by Western blot analysis (Figure 7E). The production of VEGF and HGF protein, along with the expression of EC markers CD31 and VEGFR2, was all down‐regulated to the similar level as the control (Figure 7F). Meanwhile, the effect of GDF11 on apoptosis‐related proteins cleaved caspase‐3, Bax and Bcl‐2 was also blocked by the inhibitors, indicating that the function of GDF11 on enhancing resistant of MSCs to apoptosis is also associated with phosphorylation of ERK. As shown in the schematic diagram, these results demonstrate that the TGFβ‐R/ERK/EIF4E signalling pathway plays an important role in GDF11‐induced differentiation of MSCs into ECs. These results demonstrate that the TGFβ‐R/ERK/EIF4E signalling pathway plays an important role in GDF11‐induced differentiation of MSCs into endothelial‐like cells (Figure 7G).

## 4 DISCUSSION

In this study, we showed that GDF11 and VEGF had mutual effects on differentiation of MSCs into endothelial‐like cells. GDF11 can stimulate the expression of VEGF and vice versa. Both of these molecules promoted MSC differentiation into endothelial‐like cells. Either by overexpressing it in the target cells or providing the protein directly, GDF11 was shown to boost MSC differentiation by enhancing the expression of endothelial‐related markers CD31, VEGFR2 and increasing the viability and retention rate of MSCs following implantation. MSCs overexpressing GDF11 had an enhanced ability of tube formation in vitro and promoted more vascularization in vivo. All these results suggest that GDF11 plays a critical role in differentiation and function of MSCs.

It has been almost two decades since BM‐MSCs were first used to promote angiogenesis for ischaemic diseases.^32^ BM‐MSCs were shown to be able to differentiate into ECs both in vitro^16^ and in vivo.^33^ However, the differentiation rate is low and newly formed capillaries were unstable.^34^ There are several factors influencing differentiation of stem cells into ECs. For example, endothelial growth supplements,^35^, ^36^ shear forces^37^, ^38^ and composition of extracellular matrix^39^ are important factors in EC differentiation. Some members in the TGF‐β family have been reported to promote endothelial differentiation. Bone morphogenetic protein 4 (BMP4) accelerated the commitment of human embryonic stem cells to the endothelial lineage.^40^ Treatment of iPSCs with TGF‐β2 can induce EC marker expression and in vitro tube formation.^41^ GDF11, as a member of TGF‐β family, has a similar function as other family members, such as TGF‐β1, to promote angiogenesis.^42^, ^43^ GDF11 was reported to promote migration and sprouting of endothelial progenitor cells^44^ and other angiogenic activities.^45^

Here, we show for the first time that GDF11 has direct effect on MSC differentiation into endothelial‐like cells. Great efforts have made to improve the efficiency of MSC differentiation^33^, ^36^ and to augment the therapeutic efficacy of stem cells.^46^, ^47^ We demonstrated that MSC^GDF11^ had a greater ability to differentiate into endothelial‐like cells, as evidenced by significantly higher expression of EC‐related markers at both mRNA and protein levels (Figures 1D,F and 2). The in vitro results were confirmed in vivo by implantation of MSC‐containing Matrigel plugs into mice (Figure 4A). When the Matrigel plugs with the MSCs are implanted into the subcutaneous skin of mice, either the implanted MSCs or recruited endothelial cells will form a functional vascular structure which allows blood cells in circulation come into the Matrigel plug. More angiogenesis in the Matrigel, more blood flows in, which turns the Matrigel more reddish. Our Matrigel results verified the role of GDF11 in improving the differentiation efficiency of MSCs (Figures 4 and 5 and Figure S6). Better differentiation efficiency of implanted stem cells could greatly improve the efficacy of stem cell therapy.

However, it is worthy to point out that some of TGF‐β family members may not have similar effect on MSCs. It has been reported that some of TGF‐β family promoted MSCs towards fibrosis, endothelial‐to‐mesenchymal transition (EndMT)^48^, ^49^ and osteoblastogenesis.^50^ As a fact, the role of GDF11 in ageing, cardiovascular diseases and function of ECs is still not fully understood, or sometimes controversial.^6^, ^7^, ^50^, ^51^ The cause of contradictory may be due to the difference in materials and methods used, objects of study and genetic background etc.^52^, ^53^ In our case, further study is needed to confirm that the differentiated EC‐like cells have real function in pro‐angiogenesis in an ischaemic disease model, and the newly formed vessels are stable in long term.

Multiple intracellular signalling pathways have been implicated in stem cell differentiation. These signalling pathways include MAP kinase signalling pathways (such as p38, ERK and JNK),^15^ Rho‐like GTPase signalling pathway^46^ and phosphatidylinositol‐3‐kinase/AKT signalling pathway.^38^ However, the detailed mechanisms for GDF11‐induced differentiation are still not well defined. For the function of GDF11, most studies have shown that GDF11 can activate the canonical Smad2/3 signalling pathway in different tissues or cells through binding with TGF‐β family receptor ActR II/I, leading to the formation of a heteromeric complex of phosphorylated Smad2/3with with Smad4 and then translocation to the nucleus to regulate gene expression. However, we did not observe conclusive Smad2/3 activation in MSCs by GDF11 during endothelial differentiation (Figure S8). This agrees with the previous report that roles of Smad2/3 signalling are stage‐dependent during endothelial differentiation of MSCs induced by TGF‐β1 or BMPs.^54^, ^55^

Indeed, the downstream pathways of TGF‐β receptors could be either Smad‐dependent or Smad‐independent.^13^, ^14^ It has been reported that GDF11 activates p38 MAPK to regulate the size and function of the nucleolus, affects JNK in ECs, as well as cross talking with AMPK, eNOS and NF‐κB.^8^ On the other hand, we found greater ERK phosphorylation when MSC^GDF11^ were induced for endothelial differentiation by VEGF165, as compared with the control group (Figure 6). In addition, we also detected more phosphorylated EIF4E, the downstream target of ERK pathway. These activations can be blocked by both inhibitors for TGF‐β receptor and ERK (Figure 6E). It is known that EIF4E is an important component for initiation of translation and its phosphorylation activates the translation of cellular proteins.^56^ When EIF4E was knocked down by siRNA, the translation of its downstream targets including c‐Myc, VEGF, CyclinD1 and Bcl‐2 were diminished.^57^ Our data show that activation of the ERK/EIF4E pathway was also inhibited when GDF11 was knocked down. The anti‐apoptotic effect of GDF11 on MSCs was blocked by inhibition of TGFβ‐R or ERK (Figure 7E). Together, these results suggest that the TGFβ‐R/ERK/EIF4E pathway is crucial for the actions of GDF11 in this system.

It is well established that most of growth factors usually regulate their effect via activation of the TGFβ‐RAS‐RAF‐MEK1/2‐ERK1/2 signalling cascade.^58^ We would infer that GDF11 binds to the TGF‐β receptor and subsequent activates RAS‐RAF‐MEK‐ERK/EIF4E pathway to induce the endothelial differentiation of MSC. Our results are consistent with previous reports showing that the ERK/EIF4E pathway is involved in cell growth, regulating cell cycle and apoptosis.^59^

Furthermore, we also showed that GDF11 and VEGF had mutual effects on the expression and differentiation of MSCs into endothelial‐like cells. VEGF binding with its VEGFR receptors can also activate multiple downstream pathways, like PKC‐ERK, which will enhance the expression of genes, for example GDF11, for cell differentiation and proliferation (Figure 7G). However, the specific molecular mechanism of the interaction between GDF11 and VEGFA needs further study to be reviewed.

In summary, we found that GDF11 can significantly enhance the potential of MSCs for endothelial differentiation, increase their viability, and augment the therapeutic efficacy of MSCs to promote angiogenesis. These actions of GDF11 may involve its binding to the TGF‐β receptor and subsequent activation of ERK/EIF4E pathway. This novel function of GDF11 in MSCs could be useful for stem cell therapy for ischaemic cardiovascular diseases.

## CONFLICT OF INTEREST

The authors declare that they have no conflicts of interest.

## AUTHOR CONTRIBUTION

**Chi Zhang:** Data curation (lead); Formal analysis (lead); Investigation (lead); Project administration (lead). **Yinuo Lin:** Conceptualization (lead); Data curation (lead). **Qi Liu:** Data curation (equal); Methodology (equal). **Junhua He:** Formal analysis (equal); Software (equal). **Pingping Xiang:** Investigation (equal). **Dianliang Wang:** Resources (supporting); Writing‐review & editing (supporting). **Xinyang Hu:** Resources (equal); Validation (equal). **Jinghai Chen:** Resources (equal); Visualization (equal); Writing‐original draft (equal). **Wei Zhu:** Project administration (equal); Writing‐review & editing (equal). **Hong Yu:** Project administration (lead); Software (equal); Supervision (lead); Writing‐original draft (equal); Writing‐review & editing (lead).

## Supporting information

Supplementary Material

## Data Availability

The data that support the findings of this study are available from the corresponding author upon reasonable request.
