# Supplementary material for: Growth differentiation factor 11 promotes differentiation of MSCs into endothelial‐like cells for angiogenesis
Source: J Cell Mol Med. 2020 Jun 25;24(15):8703–17. doi: 10.1111/jcmm.15502 (PMC7412688; doi:10.1111/jcmm.15502)
Supplement: Supplementary file 1 — Supplementary Material [file JCMM-24-8703-s001.docx]

**Supplementary Materials**

**GDF11 promotes differentiation of MSCs into endothelial cells for angiogenesis**

Running title: GDF11 promotes MSC differentiation into EC

Chi Zhang ^1#^, Yinuo Lin^3,1#^, Qi Liu ^1^, Junhua He ^1^, Pingping Xiang^1^, Xinyang Hu^1,2^, Jinghai Chen^1,2^, Wei Zhu^1,2^, Hong Yu ^1,2*^

1. Department of Cardiology, Second Affiliated Hospital, College of Medicine, Zhejiang University. 88 Jiefang Rd, Hangzhou, Zhejiang Province, 310009, PR China

2. Cardiovascular Key Laboratory of Zhejiang Province. 88 Jiefang Rd, Hangzhou, Zhejiang Province, 310009, PR China

3. Department of Cardiology, The First Affiliated Hospital of Wenzhou Medical University, Wenzhou, Zhejiang, China

# CZ and YL contributed equally to this article.

***Corresponding author:** Hong Yu, Ph.D., Department of Cardiology, Second Affiliated Hospital, College of Medicine, Zhejiang University, 88 Jiefang Rd, Hangzhou 310009, P.R. China. Tel: +86-571-87783992; Fax: 86-571-87037885;

E‐mail address: [yuvascular@zju.edu.cn](mailto:yuvascular@zju.edu.cn)

**Supplementary Materials**

**Supplementary Figure S1.**


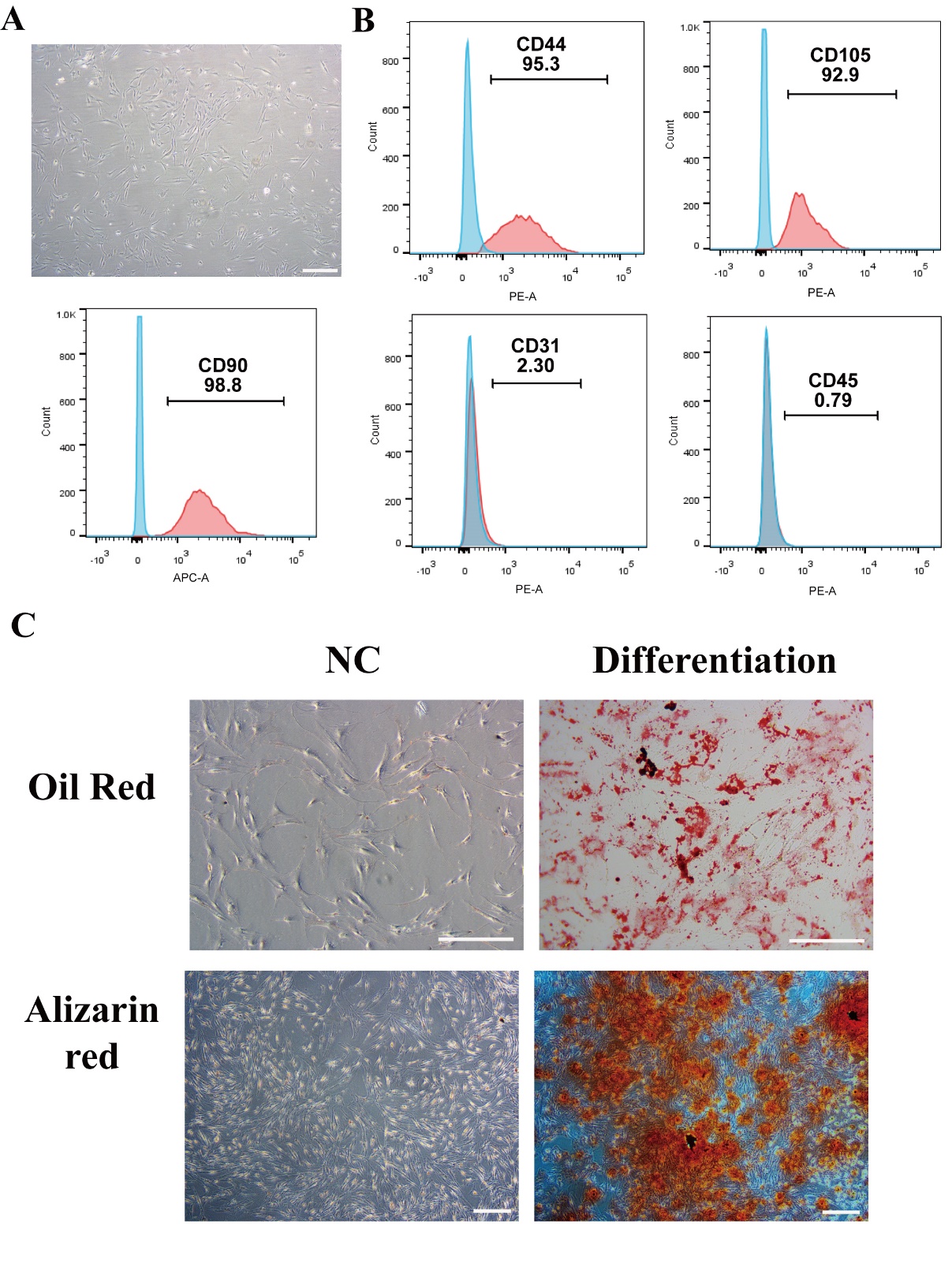


**Supplementary Figure S1. Characterization of MSCs and overexpression of GDF11.**

A) Representative images of BM-MSCs (P3) under phase contract microscopy demonstrated a spindle-shaped and fibroblast-like morphology. Scale bars=100μm.

B) Flow cytometry analysis of MSCs that were uniformly labeled with antibodies against the indicated antigens. MSCs were positive for CD44, CD105 and CD90, but negative for CD31, CD45.

C) MSCs were differentiated into Osteocytes and Adipocytes. Left panel: under phase contract microscopy; Right panel: cells were stained with oil red or Alizarin red.

**Supplementary Figure S2.**


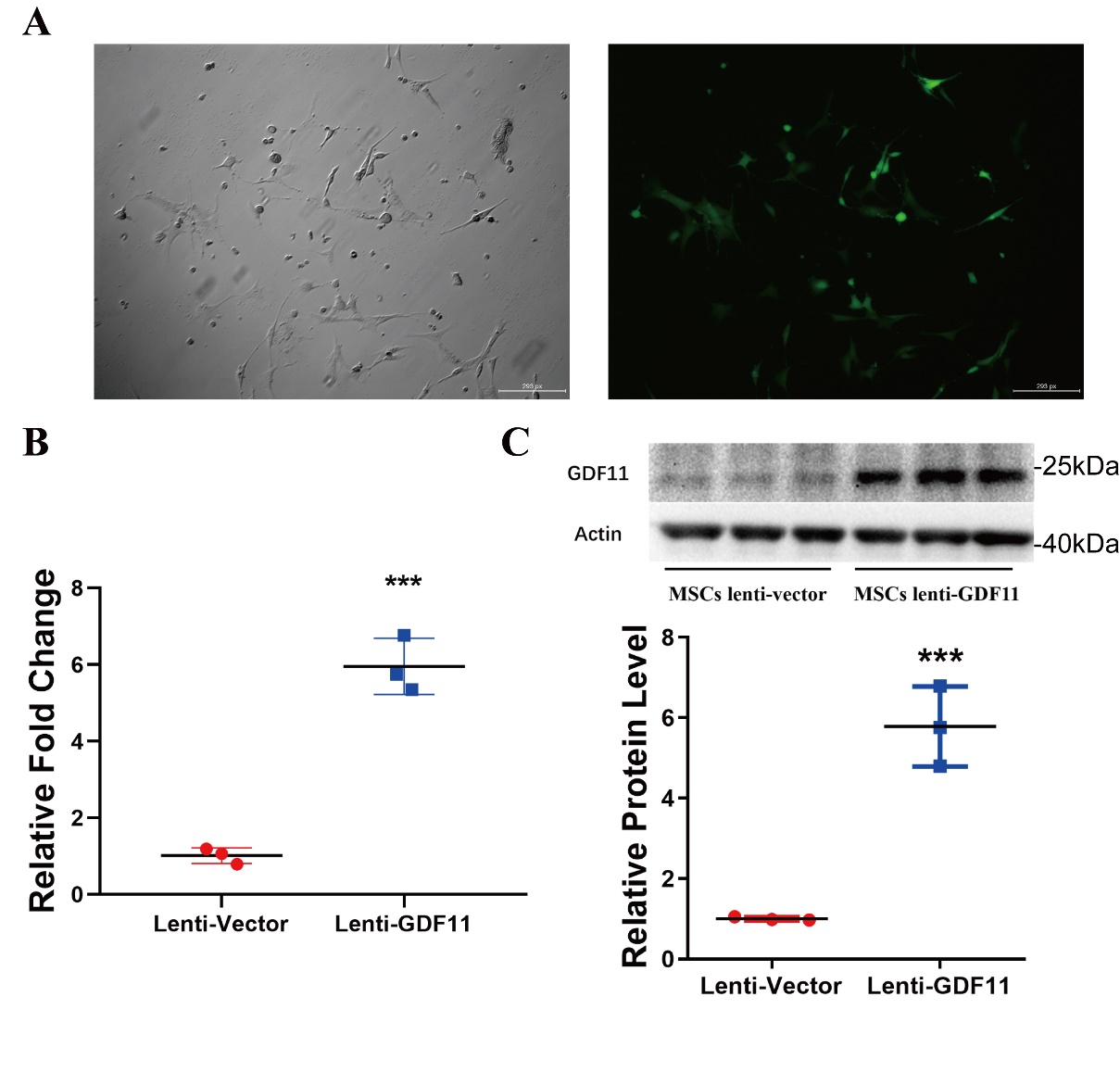


**Supplementary Figure S2. Overexpression of GDF11 in MSCs.**

A) Representative images of MSCs after they were transduced with lentiviral vector LV-GFP-GDF11. Left panel was under phase contract microscopy and Right panel was under fluorescent microscope showing GFP-labeled cells. Scale bars=50μm.

B) GDF11 expression at mRNA level was measured by real-time RT-PCR. GDF11 mRNA in MSCs^GDF11^ is higher than MSCs^Vector^. *P<0.05.

C) Western blotting analysis of GDF11 protein and its quantification. MSCs^GDF11^ had more GDF11 protein than MSCs^Vector^.

**Supplementary Figure S3.**


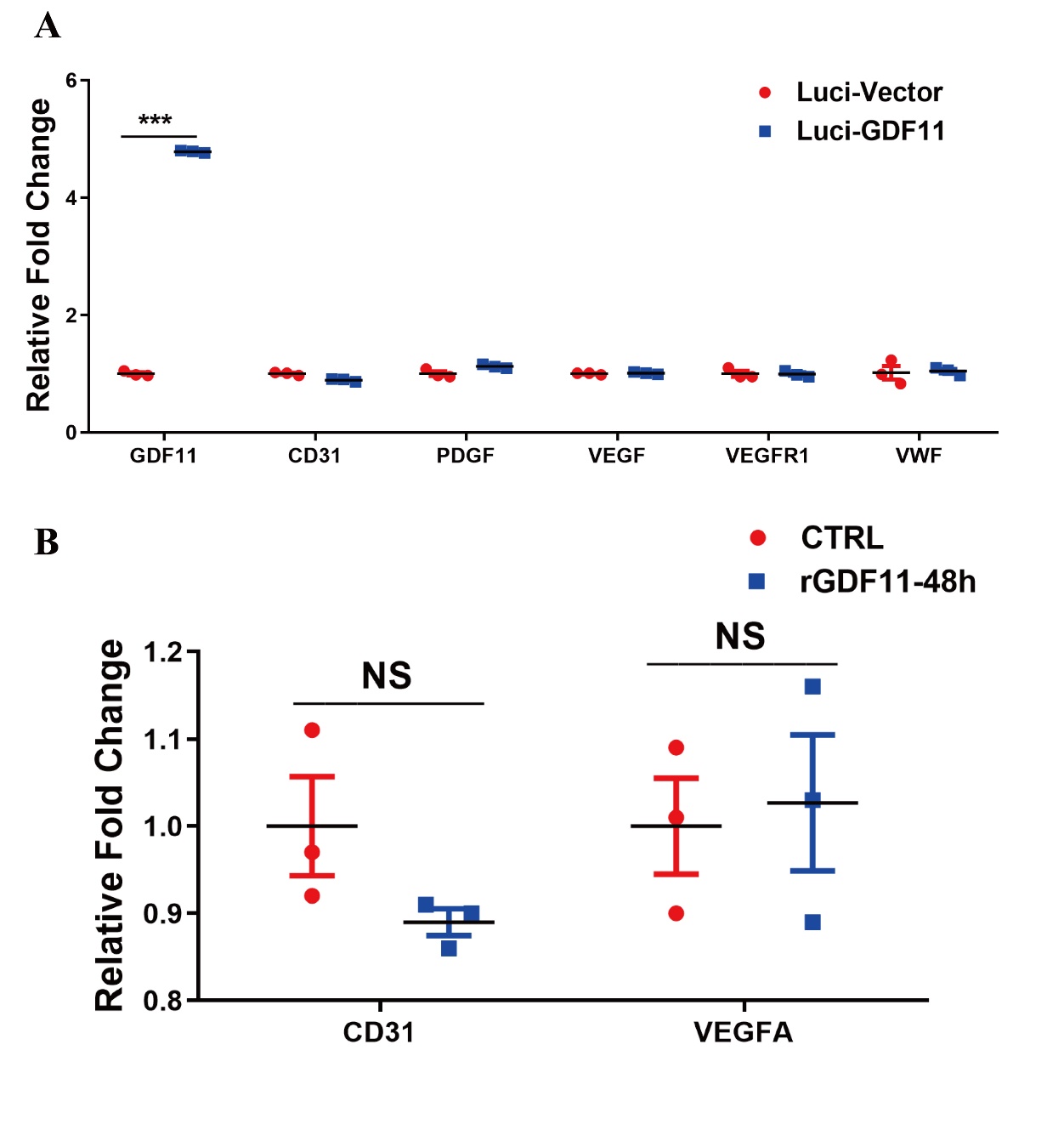


**Supplementary Figure S3. Expression of EC markers in MSCs.**

A) Real-time PCR results showed that there was no significant difference in the expressions of EC markers between MSC^Vector^ and MSC^GDF11^.

B) MSCs were treated with recombinant factor GDF11（rGDF11）at 50ng/mL for 48h, then mRNAs of VEGFA and CD31 were quantified by RT-PCR. There was no significant difference in the expressions of VEGFA and CD31 between control MSCs and rGDF11-treated MSCs.

**Supplementary Figure S4.**


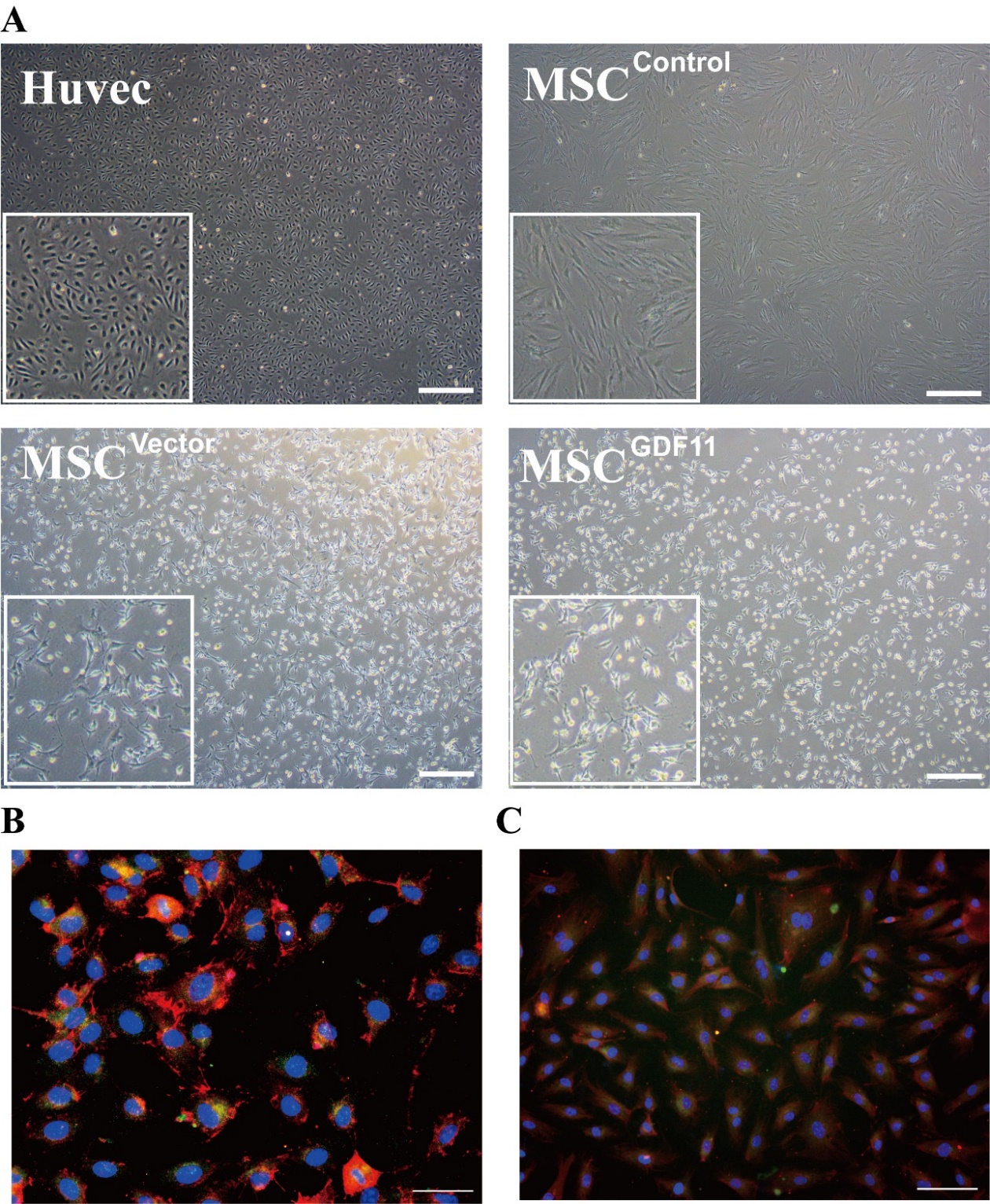


**Supplement Figure S4. GDF11 promotes MSCs to differentiate into endothelial-like cells in vitro.**

A) The morphology of cells under phase contract microscope. MSC^Vector^ and MSC^GDF11^ were cultured in the presence of VEGF165 (50 ng/ml) for 14 days to induce differentiation. HUVEC were used as positive control, MSC^control^ (upper right) without treatment by VEGF165 were used as negative control. The inserts with white border are magnified 4x. Scale bars=200μm.

B) Immunofluorescent staining of differentiated MSCs with Abs against CD31+ (red) and VEGFR2. +(green). Nucleus were stained with DAPI (blue). MSCs were cultured in the presence of VEGF165 for 14 day. Scale bars=50μm.

C) HUVEC as a positive control were stained in the same way as a positive control. Scale bars=50μm.

**Supplementary Figure S5.**


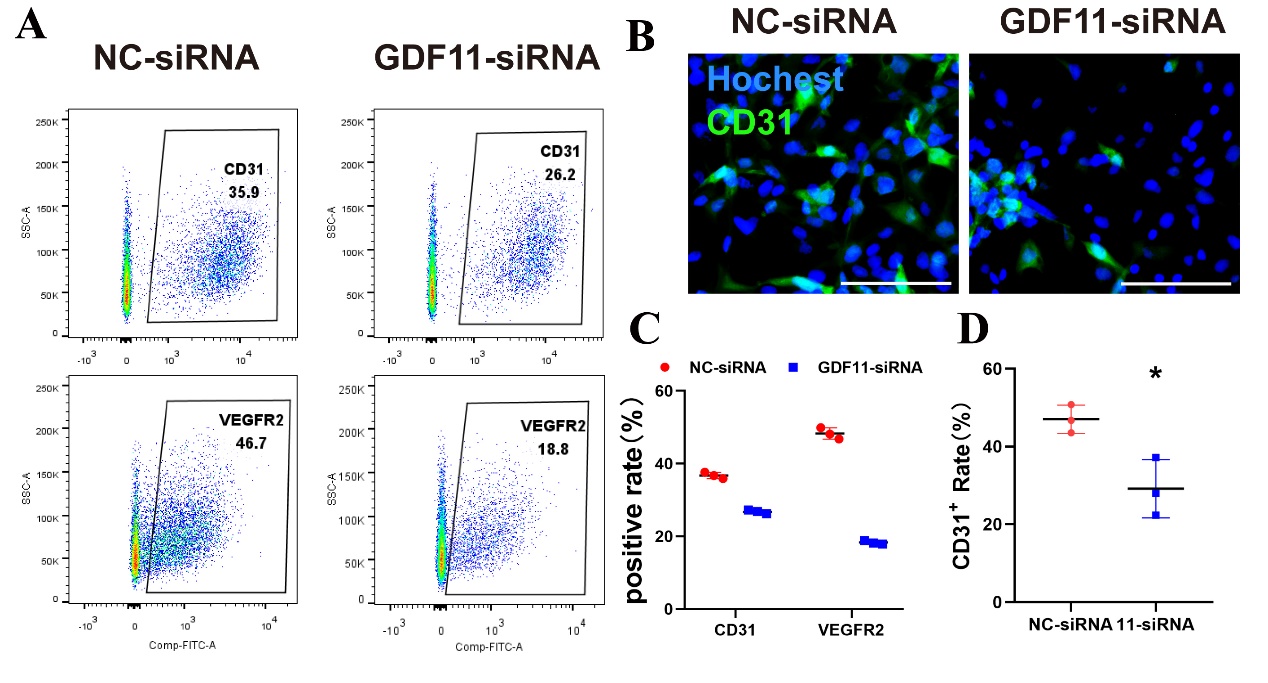


**Supplementary Figure S5. The down-expression of GDF11 in MSCs reduces the differentiation into endothelial-like cells**

A) Flow cytometry analysis of EC markers (CD31 in upper panel, VEGFR2 in lower panel) on GDF11-siRNA and NC-siRNA. B) Immunofluorescent staining of NC-siRNA and 11-siRNA with Abs against EC marker CD31 (green). Nucleus were stained with DAPI (blue). MSCs after GDF11 down-expression were cultured at the presence of VEGF165 for 14 days to induce differentiation. Scale bars=100 μm. C) Quantification of positive rates in A (n=3). D) Quantification of CD31+ cells in MSCs shown in B. *p<0.05; **p<0.01 and ***p<0.001.

**Supplementary Figure S6.**

**
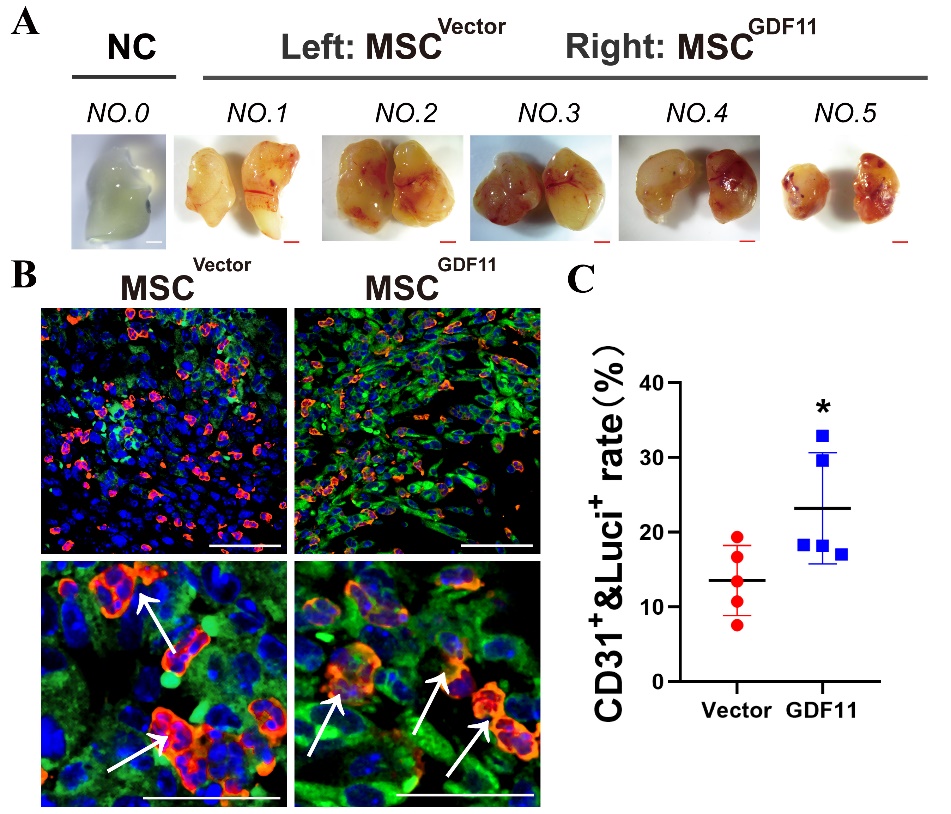
**

**Supplementary Figure S6. GDF11 promotes angiogenesis in Matrigel plug in vivo.**

Photoimages of the recovered Matrigel plugs from mice showing gross appearance of angiogenesis (n=5), NC: PBS with no cells as Negative control; Left of the paired plugs: MSCs^Vector^, right: MSCs^GDF11^, Scale bars=4 mm.

B) Antibodies against luciferase (green) and CD31 (red) for endothelial-like cells were used. White arrows point at the double positive cells (orange).

C) Rates of MSCs^Vector^ or MSCs^GDF11^ differentiation into endothelial-like cells were similarly quantified. The pictures in upper panel were taken at 600x magnification and the lower panel are magnified 2x. Scale bars: 50μm.

**Supplementary Figure S7.**


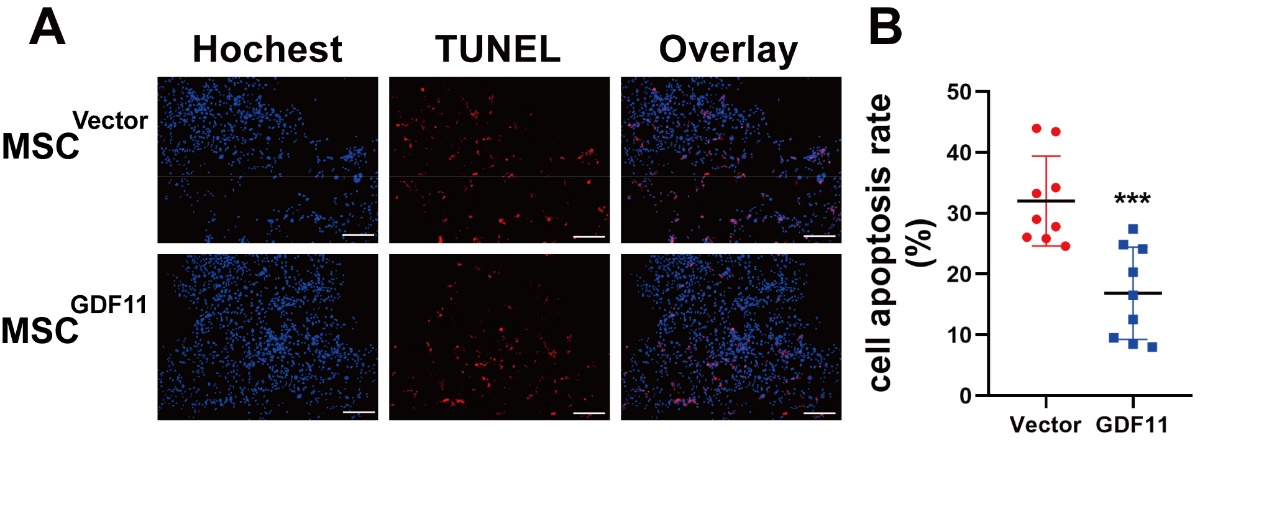


**Supplementary Figure S7. GDF11 protects MSCs through anti-apoptosis in vitro.**

A) Apoptotic MSC^Vector^ and MSC^GDF11^ were identified via TUNEL staining (red) after they were cultured under hypoxia condition. Nuclei were stained with DAPI (blue) (n=9); Scale bars=100μm.

B) Quantification of TUNEL positive cells.

**Supplementary Figure S8.**
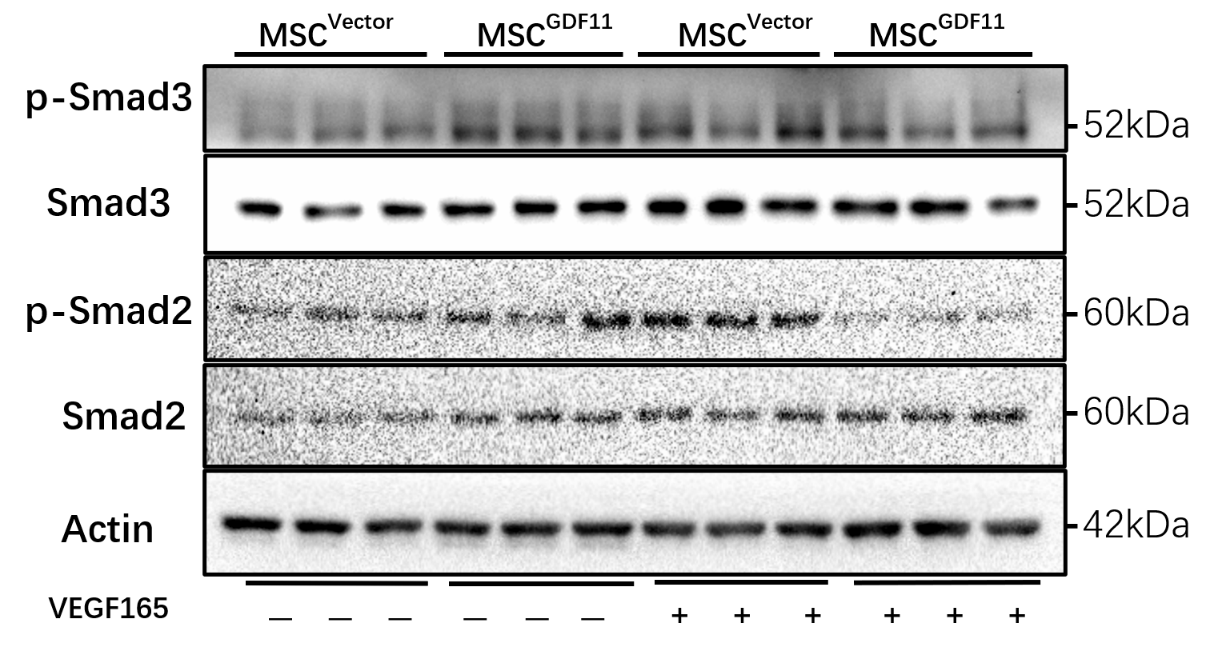


**Supplementary Figure S8. Effect of GDF11 and VEGF on phosphorylation of Smad2/3 in MSCs.** A) MSCs^Vector^ and MSC^GDF11^ were cultured in the presence or absence of VEGF165. Phosphorylation of Smad2/3 were examined by Western blot (n=3). When MSCs were cultured under differentiation conditions with VEGF165, Smad2/3 were not activated.

**Supplementary Table S1. Primer sequences for analysis of gene expression by RT-PCR.**

| Gene | Forward | Reverse |
| --- | --- | --- |
| ACTIN | GCCTTCCTTCTTGGGTATGG | GCACTGTGTTGGCATAGAGG |
| GDF11 | GAAGAGGACGAGTACCAGCG | ATCACCTTGGGGCTGAAGTG |
| CD31 | GAAGTGTCCTCCCTTGAGCC | GGAGCCTTCCGTTCTTAGGC |
| VWF | GGGTGACCAAAGCATCTCCA | CATCGATTCTGGCCGCAAAG |
| VEGF | CGTCAGAGAGCAACATCACC | GCGCTTTCGTTTTTGACCCTT |
| VEGFR1 | CTGCGACCCTCTTTTGGCTC | CAGTCTCTCCCGTGCAAACT |
| VEGFR2 | CTCTGTGGTTCTGCGTGGAG | CGCTGTCCCCTGCAAGTAAT |
| PDGF | CTCCGTAGATGAAGATGGGGC | TGGTGCGATCGATGAGGTTC |
| TGF-β | AGGGCTACCATGCCAACTTC | CCACGTAGTAGACGATGGGC |
| GDF8 | TCTTGTGCACCAAGCAAACC | CACCCACAGCGGTCTACTAC |
